# Supplementary material for: Genetic loci and metabolic states associated with murine epigenetic aging
Source: eLife. 2022 Apr 7;11:e75244. doi: 10.7554/eLife.75244 (PMC9049972; doi:10.7554/eLife.75244)
Supplement: Supplementary file 4. — (a) Sex differences in epigenetic aging after correction for body weight. (b) Pearson correlations between epigenetic age acceleration and strain-level longevity summaries. (c) High-priority candidate genes in quantitative trait locus for epigenetic age acceleration. [file elife-75244-supp4.docx]

**Supplementary Tables**

**Supplementary file 4a. Sex differences in epigenetic aging after correction for body weight**

| **Predictor** | **Outcome** | **Estimate** | **Std Error** | **t Ratio** | **p** |
| --- | --- | --- | --- | --- | --- |
| Sex[M]  lm(x ~ sex + BWF)  n = 58  (40 females, 18 males) | EAA, pan | 0.171 | 0.066 | 2.61 | 0.012 |
|  | EAA, liver | 0.098 | 0.055 | 1.79 | 0.079 |
|  | dev.EAA, pan | 0.180 | 0.059 | 3.05 | 0.004 |
|  | dev.EAA, liver | 0.176 | 0.057 | 3.07 | 0.003 |
|  | int.EAA, pan | 0.324 | 0.077 | 4.20 | 9.9E-05 |
|  | int.EAA, liver | 0.133 | 0.078 | 1.72 | 0.092 |

**Supplementary file 4b. Pearson correlations between epigenetic age acceleration and strain-level longevity summaries**

| **DNAm** | **minLS** | | **25Q-LS** | | **Mean LS** | | **Median LS** | | **75Q-LS** | | **Max LS** | |
| --- | --- | --- | --- | --- | --- | --- | --- | --- | --- | --- | --- | --- |
|  | **r** | **p** | **r** | **p** | **r** | **p** | **r** | **p** | **r** | **p** | **r** | **p** |
| EAA, pan | 0.08 | 0.15 | 0.06 | 0.27 | -0.06 | 0.34 | -0.08 | 0.17 | -0.16 | 0.005 | -0.13 | 0.02 |
| EAA, liver | -0.02 | 0.73 | -0.03 | 0.58 | -0.07 | 0.24 | -0.08 | 0.18 | -0.08 | 0.15 | -0.06 | 0.27 |
| dev.EAA, pan | -0.02 | 0.70 | 0.03 | 0.59 | -0.02 | 0.69 | -0.04 | 0.50 | -0.06 | 0.33 | -0.01 | 0.91 |
| dev.EAA, liver | -0.05 | 0.35 | -0.01 | 0.83 | -0.05 | 0.37 | -0.08 | 0.18 | -0.07 | 0.21 | 0.01 | 0.86 |
| int.EAA, pan | 0.02 | 0.73 | -0.01 | 0.91 | -0.05 | 0.35 | -0.09 | 0.12 | -0.08 | 0.17 | -0.04 | 0.48 |
| int.EAA, liver | -0.19 | 0.0007 | -0.15 | 0.01 | -0.19 | 0.001 | -0.19 | 0.001 | -0.18 | 0.002 | -0.14 | 0.02 |

n = 302 females BXDs belonging to 65 genotype-by-diet combinations with at least n = 5 observations of age at natural death.

38 BXD genotypes on normal chow, and 27 BXD genotypes on high fat diet.

**Supplementary file 4c. High priority candidate genes in QTLs for epigenetic age acceleration**

|  |  | |  | **Missense/stop variant in the BXD** | | | |  | **Human GWAS** | |
| --- | --- | --- | --- | --- | --- | --- | --- | --- | --- | --- |
| **Gene** | | **Chr** | **Mb** | **Csq ^a^** | **dbSNP ^a^** | **Ref ^a^** | ***D* ^a^** | **LOD (high allele)^b^** | **Human trait** | **Reference** |
| *Mmd* | | 11 | 90.25 | cis-eQT |  |  |  | 7.4 (B) | Menarche (age at onset) | ^1^ |
| *Stxbp4* | | 11 | 90.54 | missense; cis-eQT | rs3668623 | C | T | 6.1 (D) | Intrinsic epigenetic age acceleration; Menarche (age at onset) | ^2-4^ |
| *Tom1l1* | | 11 | 90.67 | missense; cis-eQT | rs13469307; rs13469308 | G; G | A; C | 2.7 (D) | Parental longevity (mother's age at death or mother's attained age) | ^5^ |
| *Abi3* | | 11 | 95.83 | missense | rs29392269 | G | A |  | Late onset & and family history of Alzheimer's disease | ^6,7^ |
| *Cdk12* | | 11 | 98.20 | missense | rs27086373 | C | A |  | Menopause (age at onset) | ^8^ |
| *Cyp26a1* | | 19 | 37.70 | missense | rs8236989 | G | A |  | Human longevity | ^9^ |
| *Myof* | | 19 | 38.00 | missense | rs31052565; rs46477910 | A; G | G; T |  | Human longevity | ^9^ |
| *Ccnj* | | 19 | 40.83 | missense; cis-eQT | rs36487301 | C | A | 4.1 (D) | Menopause (age at onset) | ^10^ |
| *Nkx2-3* | | 19 | 43.62 | missense | rs30898786 | T | G |  | Epigenetic age acceleration (PhenoAge) | ^11^ |
| *Cutc* | | 19 | 43.75 | cis-eQT |  |  |  | 37.6 (B) | Epigenetic age acceleration (Hannum and PhenoAge) | ^11^ |
| *Chuk* | | 19 | 44.08 | missense | rs48727905 | T | G |  | Age at menopause | ^1^ |
| *Pkd2l1* | | 19 | 44.15 | missense | rs30956598; rs13483639 | A; T | G; C |  | Parental extreme longevity (95 years and older) | ^12^ |

**^a^** Data from the Wellcome Sanger Institute Mouse Genome Project. Each gene also contains multiple non-coding variants. Ref (reference) is the allele for C57BL/6J; *D* is the allele for DBA/2J.

**^b^** LOD score for gene expression *cis*-eQTL in BXD liver. *B* means the C57BL/6J allele has the positive additive effect; *D* means the DBA/2J allele has the positive additive effect.

**Reference for Supplementary file 4c:**

1. Kichaev G, Bhatia G, Loh PR, et al. Leveraging Polygenic Functional Enrichment to Improve GWAS Power. *American journal of human genetics.* 2019;104(1):65-75.

2. Lu AT, Xue L, Salfati EL, et al. GWAS of epigenetic aging rates in blood reveals a critical role for TERT. *Nature communications.* 2018;9(1):387.

3. Perry JR, Day F, Elks CE, et al. Parent-of-origin-specific allelic associations among 106 genomic loci for age at menarche. *Nature.* 2014;514(7520):92-97.

4. Day FR, Thompson DJ, Helgason H, et al. Genomic analyses identify hundreds of variants associated with age at menarche and support a role for puberty timing in cancer risk. *Nature genetics.* 2017;49(6):834-841.

5. Wright KM, Rand KA, Kermany A, et al. A Prospective Analysis of Genetic Variants Associated with Human Lifespan. *G3.* 2019;9(9):2863-2878.

6. Sims R, van der Lee SJ, Naj AC, et al. Rare coding variants in PLCG2, ABI3, and TREM2 implicate microglial-mediated innate immunity in Alzheimer's disease. *Nature genetics.* 2017;49(9):1373-1384.

7. Jansen IE, Savage JE, Watanabe K, et al. Genome-wide meta-analysis identifies new loci and functional pathways influencing Alzheimer's disease risk. *Nature genetics.* 2019;51(3):404-413.

8. Day FR, Ruth KS, Thompson DJ, et al. Large-scale genomic analyses link reproductive aging to hypothalamic signaling, breast cancer susceptibility and BRCA1-mediated DNA repair. *Nature genetics.* 2015;47(11):1294-1303.

9. Yashin AI, Arbeev KG, Wu D, et al. Genetics of Human Longevity From Incomplete Data: New Findings From the Long Life Family Study. *The journals of gerontology Series A, Biological sciences and medical sciences.* 2018;73(11):1472-1481.

10. Horikoshi M, Day FR, Akiyama M, et al. Elucidating the genetic architecture of reproductive ageing in the Japanese population. *Nature communications.* 2018;9(1):1977.

11. McCartney DL, Min JL, Richmond RC, et al. Genome-wide association studies identify 137 loci for DNA methylation biomarkers of ageing. *bioRxiv.* 2020:2020.2006.2029.133702.

12. Pilling LC, Atkins JL, Bowman K, et al. Human longevity is influenced by many genetic variants: evidence from 75,000 UK Biobank participants. *Aging.* 2016;8(3):547-560.
