# Supplementary material for: Genetic loci and metabolic states associated with murine epigenetic aging
Source: eLife. 2022 Apr 7;11:e75244. doi: 10.7554/eLife.75244 (PMC9049972; doi:10.7554/eLife.75244)
Supplement: Transparent reporting form [file elife-75244-transrepform1.docx]

***eLife’s* transparent reporting form**

We encourage authors to provide detailed information *within their submission* to facilitate the interpretation and replication of experiments. Authors can upload supporting documentation to indicate the use of appropriate reporting guidelines for health-related research (see [EQUATOR Network](http://www.equator-network.org/%20)), life science research (see the [BioSharing Information Resource](https://biosharing.org/)), or the [ARRIVE guidelines](http://www.plosbiology.org/article/info:doi/10.1371/journal.pbio.1000412) for reporting work involving animal research. Where applicable, authors should refer to any relevant reporting standards documents in this form.

If you have any questions, please consult our Journal Policies and/or contact us: [editorial@elifesciences.org](mailto:editorial@elifesciences.org).

**Sample-size estimation**

- You should state whether an appropriate sample size was computed when the study was being designed
- You should state the statistical method of sample size computation and any required assumptions
- If no explicit power analysis was used, you should describe how you decided what sample (replicate) size (number) to use

Please outline where this information can be found within the submission (e.g., sections or figure legends), or explain why this information doesn’t apply to your submission:

This study is in a population of genetically diverse inbred mouse strains. Information on sample size and descriptions are provided in the 1^st^ paragraph of “**Results**”, and the individual-level data are in **Data S1**

We also provide sample size n in figure captions.

Description of the statistical methods can be found under “**Methods**”, and we have provided the statistical equations.

**Replicates**

- You should report how often each experiment was performed
- You should include a definition of biological versus technical replication
- The data obtained should be provided and sufficient information should be provided to indicate the number of independent biological and/or technical replicates
- If you encountered any outliers, you should describe how these were handled
- Criteria for exclusion/inclusion of data should be clearly stated
- High-throughput sequence data should be uploaded before submission, with a private link for reviewers provided (these are available from both GEO and ArrayExpress)

Please outline where this information can be found within the submission (e.g., sections or figure legends), or explain why this information doesn’t apply to your submission:

We assayed DNA methylation in close to 350 BXD mice. The quality checks, strain verification steps, and sample exclusion (if failed QC and verification) are described under “**Materials and Methods**” (line 466 on).

We provide the complete individual-level data in **Data S1**, and for additional relevant data, we provide a “How to access data” in **Data S15**. All data we used are in a FAIR+ compliant publicly accessible repository, and details are provided in the manuscript and data accession IDs are in **Data S15**.

The processed and normalized microarray data is available through a web portal (see **Data Availability**).

We will also submit the full raw data to NCBI GEO upon official publication.

**Statistical reporting**

- Statistical analysis methods should be described and justified
- Raw data should be presented in figures whenever informative to do so (typically when N per group is less than 10)
- For each experiment, you should identify the statistical tests used, exact values of N, definitions of center, methods of multiple test correction, and dispersion and precision measures (e.g., mean, median, SD, SEM, confidence intervals; and, for the major substantive results, a measure of effect size (e.g., Pearson's r, Cohen's d)
- Report exact p-values wherever possible alongside the summary statistics and 95% confidence intervals. These should be reported for all key questions and not only when the p-value is less than 0.05.

Please outline where this information can be found within the submission (e.g., sections or figure legends), or explain why this information doesn’t apply to your submission:

The statistical information are provided in relevant places in the “**Results**” section, and included in figure captions and tables and supplemental data when applicable.

(For large datasets, or papers with a very large number of statistical tests, you may upload a single table file with tests, Ns, etc., with reference to sections in the manuscript.)

**Group allocation**

- Indicate how samples were allocated into experimental groups (in the case of clinical studies, please specify allocation to treatment method); if randomization was used, please also state if restricted randomization was applied
- Indicate if masking was used during group allocation, data collection and/or data analysis

Please outline where this information can be found within the submission (e.g., sections or figure legends), or explain why this information doesn’t apply to your submission:

The main grouping in this study is whether animals were on normal laboratory chow diet or on high fat diet. Animals assigned to the two diets were balanced and matched by strain. We obtained the DNA from a subset of the cohort described in PMID 34666007 and PMID 34552269, and details on the assignment to diets can be found in these two publications, and we also provide a brief description in the current manuscript under “**Materials and Methods”**.

**Additional data files (“source data”)**

- We encourage you to upload relevant additional data files, such as numerical data that are represented as a graph in a figure, or as a summary table
- Where provided, these should be in the most useful format, and they can be uploaded as “Source data” files linked to a main figure or table
- Include model definition files including the full list of parameters used
- Include code used for data analysis (e.g., R, MatLab)
- Avoid stating that data files are “available upon request”

Please indicate the figures or tables for which source data files have been provided:

More data provided in the supplemental Excel file (**Data S1 to S15**). With the exception of the raw microarray (which will eventually be deposited to NCBI GEO), all the other data we used are available to reviewers and readers.
